# Mesenchymal Stem Cells Reduce Murine Atherosclerosis Development

**DOI:** 10.1038/srep15559

**Published:** 2015-10-22

**Authors:** Vanessa Frodermann, Janine van Duijn, Melissa van Pel, Peter J. van Santbrink, Ilze Bot, Johan Kuiper, Saskia C. A. de Jager

**Affiliations:** 1 Division of Biopharmaceutics, Leiden Academic Centre for Drug Research, Leiden University, Leiden, The Netherlands; 2 Department of Immunohematology & Blood Transfusion, Leiden University Medical Center, Leiden, The Netherlands; 3 Laboratory of Experimental Cardiology, University Medical Center Utrecht, Utrecht, The Netherlands

## Abstract

Mesenchymal stem cells (MSCs) have regenerative properties, but recently they were also found to have immunomodulatory capacities. We therefore investigated whether MSCs could reduce atherosclerosis, which is determined by dyslipidaemia and chronic inflammation. We adoptively transferred MSCs into low-density lipoprotein-receptor knockout mice and put these on a Western-type diet to induce atherosclerosis. Initially after treatment, we found higher levels of circulating regulatory T cells. In the long-term, overall numbers of effector T cells were reduced by MSC treatment. Moreover, MSC-treated mice displayed a significant 33% reduction in circulating monocytes and a 77% reduction of serum CCL2 levels. Most strikingly, we found a previously unappreciated effect on lipid metabolism. Serum cholesterol was reduced by 33%, due to reduced very low-density lipoprotein levels, likely a result of reduced *de novo* hepatic lipogenesis as determined by a reduced expression of Stearoyl-CoA desaturase-1 and lipoprotein lipase. MSCs significantly affected lesion development, which was reduced by 33% in the aortic root. These lesions contained 56% less macrophages and showed a 61% reduction in T cell numbers. We show here for the first time that MSC treatment affects not only inflammatory responses but also significantly reduces dyslipidaemia in mice. This makes MSCs a potent candidate for atherosclerosis therapies.

Mesenchymal stem cells (MSCs), also called bone marrow stromal cells or mesenchymal stromal cells, are multipotent cells that can give rise to cells of the mesodermal lineage, including adipocytes, osteocytes and myocytes^1^. They were first identified in the bone marrow, but can be isolated from other tissues such as umbilical cord, placenta and adipose tissue^2^. After isolation, MSCs can be easily expanded without losing their multipotency which renders them an interesting tool for therapeutic strategies^1^. MSCs were initially investigated for their ability to repair injured heart tissue after myocardial infarction^3^,^4^,^5^. They can migrate to sites of tissue damage and inflammation, where they can extravasate, engraft the tissues and reduce scar formation^4^,^6^,^7^,^8^,^9^.

In recent years the immunomodulatory capacity of MSCs has been increasingly appreciated. Several studies have investigated the capacity of MSCs to modulate both innate and adaptive immune responses^2^,^10^,^11^,^12^,^13^,^14^,^15^. For instance, MSCs have been shown to reduce monocyte responses after myocardial infarction^11^ and to skew macrophages to an anti-inflammatory IL-10-producing phenotype^11^,^12^,^13^,^14^. MSCs also inhibit the differentiation and maturation of dendritic cells (DCs)^15^, by reducing the expression of co-stimulatory molecules and pro-inflammatory cytokines (TNF-α and IL-12), while increasing the production of anti-inflammatory cytokines (TGF-β and IL-10)^16^,^17^, which indirectly suppresses T cell proliferation^17^. However, MSCs can also directly inhibit T cell proliferation^16^,^17^,^18^, by inducing cell cycle arrest in all subsets, resulting in a quiescent state and decreased proliferation^19^.

Inflammatory processes play a crucial role in all stages of atherosclerosis. Early in the disease process, entrapped oxidized low-density lipoprotein (oxLDL) in the vessel wall leads to the activation of arterial endothelial cells and an ensuing recruitment of monocytes and T cells^20^. Upon recruitment, monocytes can differentiate to macrophages^21^. Macrophages can primarily promote T cell responses to local antigens, while DCs can activate naïve T cells in response to lesion-derived antigens in draining lymph nodes^22^. DCs are also present within lesions or can arise from blood-derived precursors. Both macrophages and DCs express scavenger receptors enabling the uptake of oxLDL and foam cell formation and toll-like receptors, which mediate activation of the antigen-presenting cells and production of pro-inflammatory cytokines. CD4^+^ T cells are crucially involved in the pathogenesis of atherosclerosis and their depletion results in a 70% reduction of lesion size^23^. The predominant subset in both human and murine atherosclerotic lesions is the Th1 subset^24^,^25^, which produces a plethora of pro-inflammatory cytokines such as IFN-γ. IFN-γ promotes vascular inflammation by enhancing maturation and activation of antigen-presenting cells, increasing macrophage lipid uptake, reducing collagen production by smooth muscle cells, and enhancing expression of endothelial adhesion molecules, which subsequently stimulates leukocyte recruitment to the lesions^26^. The continuous recruitment of further leukocytes to atherosclerotic lesions results in a vicious self-maintaining progressive inflammatory cycle. Nonetheless, it has been shown that by altering the phenotype of macrophages and DCs, they can become atheroprotective^22^,^27^,^28^. Moreover, regulatory T cells (Tregs) have been clearly established as anti-atherogenic^29^. Tregs produce high amounts of the anti-inflammatory cytokine IL-10 and inhibit ongoing inflammation.

Due to the key role of inflammatory processes in the initiation and progression of atherosclerosis, adoptive transfer of MSCs, which have the capacity to modulate and reduce inflammation, may be a therapeutic approach to treat atherosclerosis. Preclinical studies have already shown that adoptively transferred MSCs are capable of modulating immune responses and they can prevent allograft rejection^18^,^30^ and alleviate autoimmune diseases^31^,^32^,^33^. Moreover, in a phase II clinical trial, it was found that MSCs can reduce graft-versus-host disease^34^. We now show to our knowledge for the first time that MSCs in addition to their immunomodulatory capacity can significantly reduce dyslipidemia, thus establishing the multifactorial therapeutic potential of MSCs to inhibit atherogenesis.

## Results

**Figure 1:**
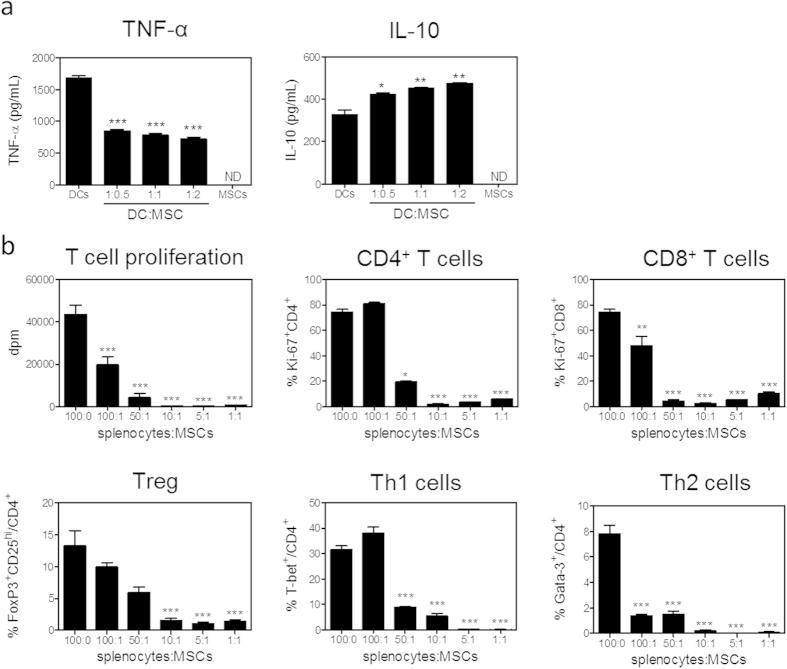
MSCs affect innate and adaptive immune responses. (**a**) DCs were co-cultured with indicated ratios of MSCs for 3 hours prior to addition of 100 ng/mL LPS for 24 hours. DCs numbers remained constant. Cytokine responses were determined by ELISA. (**b**) Splenocytes from an LDLr KO mouse were co-cultured with indicated ratios of MSCs in the presence of αCD3/CD28 for 72 hours. Proliferation was assessed by ^3^H-thymidine incorporation and Ki-67 expression by flow cytometry. Splenocyte numbers remained constant. All values are expressed as mean ± SEM and representative of at least two independent experiments done in triplicate. *P < 0.05, **P < 0.01, ***P < 0.001. ND defines not determined.

MSCs were generated from the bone marrow of male C57BL/6 mice and their phenotype was confirmed by flow cytometry analysis. All MSCs expressed Sca-1, CD29, CD44, CD105 and CD106, while CD45, CD31 and TER119 were not expressed (Supplementary Fig. 1). To confirm their immunomodulatory capacity, MSCs were co-cultured with DCs for three hours at different ratios, and subsequently stimulated with LPS. Twenty-four hours after LPS stimulation, we did not observe effects of MSCs on the expression of the co-stimulatory molecules CD40, OX40L and CD30L by DCs. The percentage of DCs positive for the co-stimulatory molecule CD86 and the negative co-stimulatory molecule PD-L2 increased by 12% and 22%, respectively. The mean expression of CD86 per cell was not significantly affected, whereas the mean expression of PD-L2 was decreased by 18% at higher MSC to DC ratios. The mean expression of the co-stimulatory molecule CD80 on DCs was significantly increased by 34% (Table 1; P < 0.001) upon co-culture with MSCs. CD80/CD86 function to induce T cell activation but can also promote Treg development, while PD-L2 functions to inhibit T cell activation, indicating that DCs might adopt a more tolerogenic phenotype after exposure to MSCs. In line with this, MSCs significantly affected the cytokine production of DCs in response to LPS. Pro-inflammatory TNF-α release was reduced by 57% (P < 0.001) while anti-inflammatory IL-10 production was increased by 45% (Fig. 1a; P < 0.01).

Since MSCs have been shown to affect T cell responses, we co-cultured MSCs with splenocytes from an atherosclerosis-prone LDLr KO mouse in the presence of αCD3/CD28 for 72 hours. MSCs potently inhibited T cell proliferation by 99%, as measured by ^3^H-thymidine incorporation, compared to αCD3/CD28 stimulated splenocytes in the absence of MSCs. CD4^+^ T cell proliferation was reduced by 92% and CD8^+^ T cell proliferation by 87%, as measured by Ki-67 expression. Polarization of all CD4^+^ T cell subsets was inhibited to a similar extent (Th1 and Th2 more than 99% and Tregs by 89%; Fig. 1b). Cytokine responses in the splenocyte co-culture indicated similar effects: IL-10 production was not detectable, IFN-γ was reduced by 99%, and TNF-α showed a 92% reduction upon the co-incubation with MSCs. Co-cultures of isolated CD4^+^ T cells and MSCs showed comparable, but milder effects: proliferation of CD4^+^ T cells was on average reduced by 80%, the skewing towards specific T cell subsets was reduced by an average of 85%, and cytokine production was also almost completely abolished (Supplementary Fig. 2). Interestingly, MSC culture supernatant had no effect on T cell proliferation, suggesting that cell-cell contact is crucial (Supplementary Fig. 3).

Before determining the effect of MSCs on atherosclerosis, we fluorescently labelled MSCs with CFSE and established the fate of these fluorescently labelled MSCs after *intravenous* injection into LDLr KO mice on a cholesterol rich diet (WTD) and determined to which organs they migrated (Supplementary Fig. 4). After fifteen minutes, MSCs accumulated primarily in the lungs and then slowly migrated out of the lungs. One to three hours after injection, we found that MSCs had migrated to the liver, the heart, the draining lymph nodes of the heart and the aorta. Surprisingly, only few MSCs were recovered in the spleen.

**Figure 2:**
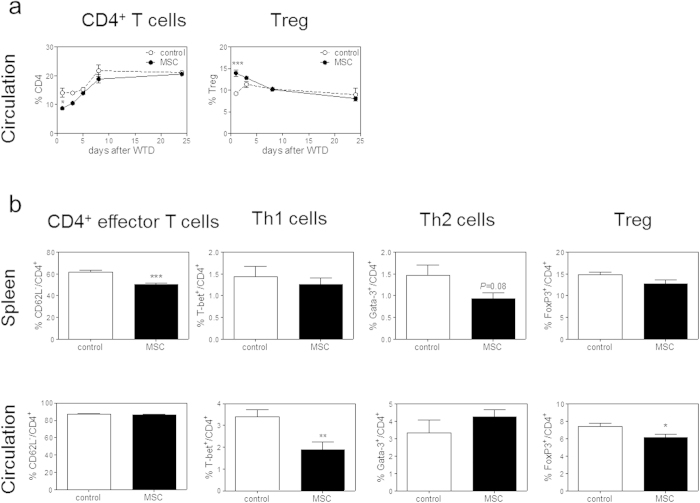
MSC treatment affects CD4^+^ T cell responses *in vivo*. Male LDLr KO mice received three *i.v.* injections of either PBS (control) or 0.5 × 10^6^ MSCs (MSC) and were then fed a Western-type diet (WTD) for eight weeks. (**a**) CD4^+^ T cells, as well as the percentage of FoxP3^+^ regulatory T cells (Treg) within CD4^+^ T cells, were measured in the circulation throughout the entire experiment by flow cytometry. (**b**) After eight weeks, effector CD4^+^ T cells, determined as CD62L^−^ within CD4^+^ T cells, as well as T cell subsets of CD4^+^ T cells in the circulation and spleen were determined by flow cytometry. All values are expressed as mean ± SEM and representative of six mice per group. *P < 0.05, **P < 0.01, ***P < 0.001.

**Figure 3:**
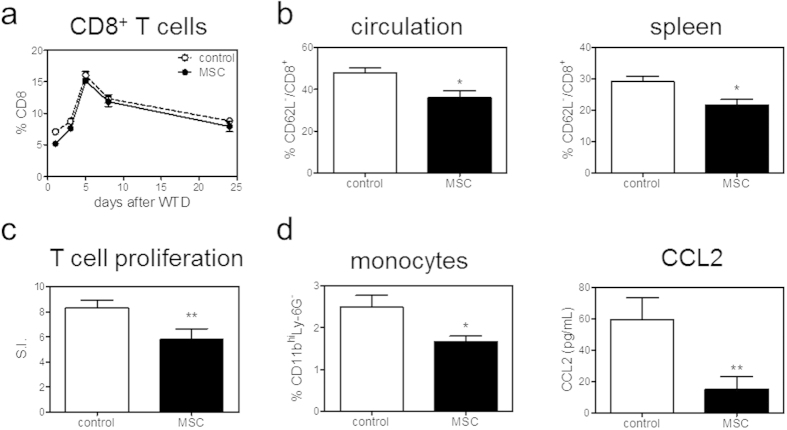
MSC treatment affects CD8^+^ T cell and monocyte responses *in vivo*. (**a**) CD8^+^ T cells were measured in the circulation throughout the entire experiment by flow cytometry. (**b**) After eight weeks, effector CD8^+^ T cells, determined as CD62L^−^ within CD8^+^ T cells, in the circulation and spleen were determined by flow cytometry. (**c**) Splenocytes were isolated and stimulated with αCD3/CD28 for 72 hours. Proliferation was assessed by the amount of ^3^H-thymidine incorporation. Proliferation is normalized for proliferation of controls (without stimulation) and expressed as the stimulation index (S.I.). (**d**) Circulating monocytes, determined as CD11b^hi^Ly-6G^−^, were analyzed by flow cytometry. CCL2 levels in serum were determined by ELISA. All values are expressed as mean ± SEM and representative of six mice per group. *P < 0.05, **P < 0.01.

**Figure 4:**
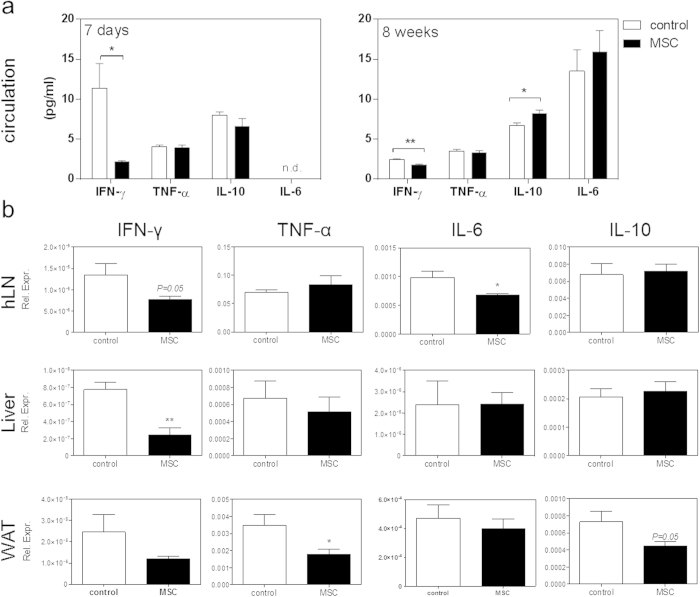
MSC treatment reduces pro-inflammatory cytokine response. (**a**) Serum IL-6 levels were measured by ELISA, while IFNγ, TNFα and IL-10 levels were determined by luminex immunoassay. mRNA expression of IL-6, TNF-α, IFN-γ and IL-10 are shown for draining lymph nodes of the heart (hLN), liver and white adipose tissue (WAT). Expression is shown relative to the expression of three housekeeping genes (SDHA, HPRT and Rpl27). All values are expressed as mean ± SEM. Serum cytokine levels are representative of n = 3 pooled samples (7 days), n = 3 control and n = 6 MSC treated (8 weeks) and mRNA cytokine expression is representative of all mice. *P < 0.05. **P < 0.01.

To test whether MSCs are able to modulate immune responses and thereby atherosclerosis, LDLr KO mice were treated with three *intravenous* injections of MSCs every other day prior to induction of atherosclerosis by WTD feeding. One day after the start of WTD, we found a significant 38% drop in circulating CD4^+^ T cells (control: 14.1% versus MSC: 8.7%, P < 0.05). Interestingly, we also observed an initial 51% increase in circulating Tregs (control: 9.2% versus MSC: 13.9%, P < 0.001; Fig. 2a). Eight weeks after inducing atherosclerosis, no difference in absolute white blood cell counts (data not shown) and percentage of circulating and splenic CD4^+^ T cells was found (Supplementary Fig. 5). A significant 18% decrease in splenic effector CD62L^−^/CD4^+^ T cells was observed (P < 0.001; Fig. 2b), while no effects on T cell subsets could be observed. In the circulation Th1 cells were significantly reduced by 44% (P < 0.01) and Tregs were reduced by 10% (P < 0.05). No significant effects of MSC therapy on Th2 cells were observed (Fig. 2b). Total CD8^+^ T cell numbers were not affected by MSC treatment in the circulation throughout the entire experiment (Fig. 3a) and were also not affected in the spleen after eight weeks of WTD (Supplementary Fig. 5). However, we again found a significant 25% decrease in effector CD62L^−^/CD8^+^ T cells in the circulation and spleen after eight weeks WTD upon MSC treatment (P < 0.05, Fig. 3b). To evaluate the proliferative capacity of T cells after MSC treatment, we isolated splenocytes eight weeks after induction of atherosclerosis by WTD feeding and cultured them in the presence of αCD3/CD28. In line with the *in vitro* data, splenocytes obtained from MSC-treated recipients showed a significant 30% decrease in T cell proliferation (P < 0.01, Fig. 3c). Furthermore, MSC treatment also significantly reduced circulating monocytes by 33% (P < 0.05), which was directly associated with a 77% reduction in serum CCL2 levels (P < 0.01; Fig. 3d), again suggesting a reduced inflammatory status of the MSC-treated mice. In line, although levels are just detectable, we observe a significant decrease in circulating IFN-γ both at 7 days (P < 0.01) and 8 weeks (P < 0.05) of WTD and a significant increase in circulating IL-10 levels (p < 0.05) in MSC treated animals after 8 weeks of WTD (Fig. 4a). Additionally, we observed a 20% reduction of IFN-γ (P = 0.05) and a 31% reduction of IL-6 (P < 0.05) in the draining lymph nodes of the heart. Moreover, we see a significant 76 % reduction of IFN-γ (P < 0.01) in the liver, and a 33% reduction of TNF-α expression (P < 0.05) in white adipose tissue (Fig. 4b).

**Figure 5:**
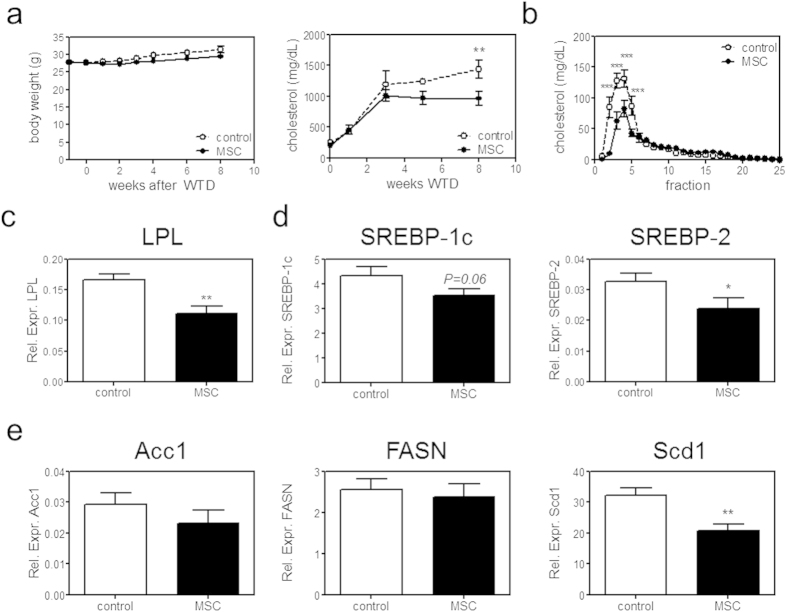
MSC treatment reduces VLDL production. (**a**) Body weight and cholesterol levels were monitored throughout the entire experiment. (**b**) Cholesterol distribution among plasma lipoprotein subclasses was determined by FPLC analysis after eight weeks WTD. For FPLC analysis serum of three mice was pooled. (**c**) Liver mRNA expression of lipoprotein lipase (LPL), (**d**) of SREBP-1c and SREBP-2, and (**e**) of acetyl-CoA carboxylase (Acc1), fatty acid synthase (FASN), stearoyl-CoA desaturase-1 (Scd1) is shown, relative to the expression of three housekeeping genes (SDHA, HPRT and Rpl27). All values are expressed as mean ± SEM and representative of all mice. *P < 0.05, **P < 0.01, ***P < 0.001.

As atherosclerosis is further determined by dyslipidaemia, we monitored weight and cholesterol levels throughout the entire experiment. Interestingly, although we did not observe a significant effect on weight or a direct effect of MSCs on plasma cholesterol levels after adoptive transfer, we found a significant 33% reduction in serum cholesterol levels in MSC-treated mice after eight weeks WTD (Fig. 5a). The decrease in cholesterol level was mainly a result of a decreased very low-density lipoprotein (VLDL) level as assessed by fast protein liquid chromatography analysis (P < 0.01; Fig. 5b). We found that the expression of hepatic lipoprotein lipase (LPL), which hydrolyses triglycerides into free fatty acids needed for VLDL synthesis, was significantly reduced by 34% (P < 0.01; Fig. 5c). In adult liver LPL is expressed by macrophages (Kupffer cells), which were not affected in their number as assessed by F4/80 and Clec4f expression, as well as CD68 expression (Supplementary Fig. 6). In white adipose tissue LPL regulates fatty acid uptake from triglyceride-rich lipoproteins for storage^35^. We found no difference in LPL expression in white adipose tissue (Supplementary Fig. 6), indicating that the reduction of LPL is specific for the liver.

Furthermore, we found a significant 27% reduction of Sterol regulatory element-binding protein (SREBP)-2 (P < 0.05) and a non-significant 18% reduction of SREBP-1c (P = 0.06; Fig. 5d); transcription factors activating multiple genes to promote triglyceride and cholesterol synthesis and thereby VLDL synthesis. Several genes involved in hepatic *de novo* lipogenesis, such as acetyl-CoA carboxylase 1 (ACC1) and fatty acid synthase (FASN) were not affected (Fig. 5e). Also diglyceride acyltransferase 1 (DGAT1), involved in the last steps of triglyceride synthesis, and microsomal triglyceride transfer protein (MTTP), important for lipoprotein assembly, were not affected (Supplementary Fig. 6). However, strikingly, the rate-limiting enzyme in fatty acid synthesis stearoyl-CoA desaturase-1 (Scd1) was significantly reduced by 35% (P < 0.01; Fig. 5e). It should be noted that while expression levels are not necessarily identical to protein levels, these are predictive for VLDL metabolism and have been shown to correlate with atherosclerosis development^36^,^37^.

**Figure 6:**
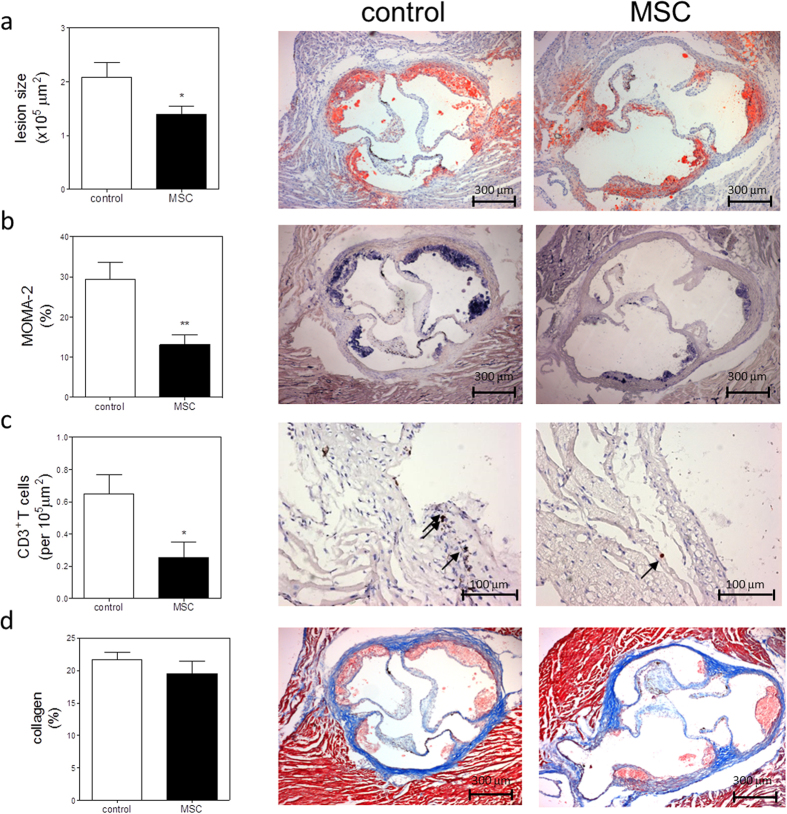
MSC treatment reduces lesion development. (**a**) Lesion size in the three valve area of the aortic root was determined; representative cross-sections stained with Oil-Red-O and hematoxylin are shown. (**b**) Macrophage content was determined by MOMA-2 staining as percentage of total lesion area. (**c**) Adventitial T cell numbers were determined by αCD3 staining. (**d**) Collagen content was determined by Masson’s Trichrome staining as percentage of total lesion area. Values are expressed as mean ± SEM and representative of all mice. *P < 0.05, **P < 0.01.

The beneficial effects of MSC treatment both on immune responses and cholesterol metabolism resulted in a significant 33% decrease in atherosclerotic lesion size in MSC-treated mice (1.4 × 10^5^ ± 0.2 × 10^5^ μm), compared with control mice (2.1 × 10^5^ ± 0.3 × 10^5^ μm, P < 0.05; Fig. 6a). Similar trends were observed when determining lesion area in all mice as percentage of vessel lumen, while lipid deposition was not affected (Supplementary Fig. 7). Additionally, we determined a significant 56% reduction in relative macrophage positive area of total lesion size (control: 29.4 ± 4.3% vs. MSC: 13.0 ± 2.6%, P < 0.01; Fig. 6b) and a 61% reduction of adventitial CD3^+^ T cells (control: 0.65 ± 0.12 cells per 10^5^ μm^2^ vs. MSC: 0.25 ± 0.09 cells per 10^5^ μm^2^, P < 0.01; Fig. 6c), indicating a reduced inflammatory status of the lesions. Lesion stability was not affected as the collagen content, determined by Masson’s trichrome staining, was not significantly different between the groups (control: 21.7 ± 1.1% vs. MSC: 19.4 ± 2.0%; Fig. 6d). Moreover, no difference in fibrous cap size and necrotic core size was observed (Supplementary Fig. 7), indicating that overall lesion composition was not affected.

## Discussion

Here we show that MSC treatment may be a promising strategy to reduce atherosclerotic lesion development. In agreement with previous studies^1^,^16^,^18^,^19^, we describe that MSCs can reduce the production of pro-inflammatory cytokines by DCs and dramatically inhibit T cell proliferation *in vitro*. We further show that adoptive transfer of MSCs into LDLr KO mice results in an overall reduced inflammatory state, as measured by reduced circulating IFN-γ and increased IL-10 levels. In addition, we observe reduced IL-6 expression in draining lymph nodes of the heart, reduced TNF-α expression in white adipose tissue and reduced IFN-γ expression in livers of MSC-treated mice. Moreover, MSC treatment results in an initial drop in circulating CD4^+^ T cell numbers. While this reduction was only observed initially after MSC transfer, a significant decrease in effector CD62L^−^/CD4^+^ and CD62L^−^/CD8^+^ T cells, as well as a significant decreased T cell proliferative capacity, was observed after eight weeks WTD in the spleen, clearly indicating a reduced differentiation of naïve T cells, which is consistent with effects observed *in vitro*. In agreement, a significant decrease in circulating Th1 cells and in Tregs was observed eight weeks after MSC transfer, as well as a significant reduction in adventitial CD3^+^ T cell numbers. However, directly after MSC treatment we observed an initial increase in circulating Tregs. This is in agreement with a recent study by Wang *et al.*^38^ who found that MSCs increased Tregs and thereby protect from atherosclerosis. However, in this study ApoE KO mice were treated multiple times with MSCs and with much higher numbers of MSCs (10^7^) compared to our study (0.5 × 10^6^), which ensured a longer increase of Tregs compared to our study. Since we observed an overall significant inhibition of T cell differentiation both *in vivo* and *in vitro*, we hypothesized that additional processes and cell types are needed for the observed increase in Tregs. It has been suggested that MSCs can induce apoptosis of T cells, which may explain the initial drop in CD4^+^ T cells that we observed *in vivo*, and that these apoptotic T cells are cleared by phagocytes, which in turn may induce Tregs^39^. Therefore *in vivo* Tregs could initially be induced indirectly, while later the overall inhibition of T cell differentiation reduces Treg numbers. Additionally, MSC therapy significantly reduced circulating monocytes and serum CCL2 levels, again clearly suggesting reduced immune responses. This is in line with a previous study showing reduced monocytes after MSC therapy for myocardial infarction^11^. The reduced monocytes and their reduced recruitment to the lesions resulted in a dramatic 56% reduction in lesional macrophages.

Unexpectedly, we found significantly lower plasma cholesterol levels in MSC-treated mice, due to a reduction of VLDL levels, which is to our knowledge an effect that was not been previously described upon MSC treatment. The effects of MSC therapy on plasma cholesterol levels only emerged around four to five weeks after treatment indicating that the effects of MSCs could be rather indirect, e.g. by modulation of other cell types. Previous studies have found a connection between immune cells and cholesterol metabolism. For example lymphotoxins, which are expressed by CD4^+^ T cells and DCs, play a role in the homeostasis of these cells and can also contribute to metabolic disease^40^,^41^. CD4^+^ T cell expression of LIGHT, another ligand of the lymphotoxin receptor, increases plasma cholesterol levels, again showing a direct link between immune cells and cholesterol metabolism^40^. Furthermore, an increased lifespan of DCs^42^ and the adoptive transfer of mature DCs was found to correlate with decreased serum cholesterol levels^43^. Hence a modulation of immune cells by MSCs could also indirectly affect cholesterol metabolism. Interestingly, we find a significant reduction of LPL expression only in the liver and not in white adipose tissue. In the adult liver, LPL is exclusively expressed by the Kupffer cells^44^. As no difference in Kupffer cell markers was found, we can assume that LPL expression is specifically decreased by the Kupffer cells themselves. While LPL KO mice develop severe hypertriglyceridemia^45^,^46^, liver-specific LPL overexpression also results in hypertriglyceridemia^47^. Moreover, LPL deficiency in macrophages has been found to reduce their uptake of VLDL or oxLDL, thereby decreasing atherosclerosis^48^,^49^. Interestingly, TNF-α has been shown to induce LPL mRNA and activity in livers in several studies^47^, indicating that reduced LPL expression could be a direct result of reduced inflammation, as we see reduced TNF-α in white adipose tissue. However, it must me noted that, although levels are very low, we do not observe an effect of MSC treatment on circulating TNF-α levels. This suggests that indeed a modulation of macrophage function by MSC treatment could have occurred. Wang *et al.*^38^ described that foam cell formation is inhibited by MSCs, which suggests that MSC can indeed directly modulate macrophage function and activation in atherosclerosis. Interestingly, LPL is secreted and results in the breakdown of triglycerides into free fatty acids, which could result in less availability of free fatty acids for VLDL synthesis by hepatocytes. In addition, previous research has found that activated Kupffer cells can express mediators that promote VLDL secretion by hepatocytes^50^, indicating that a reduced activation of Kupffer cells in MSC-treated mice could result in reduced VLDL metabolism. However, also the reduced inflammatory environment could have directly affected VLDL synthesis. In line with this, TNF-α, which is downregulated upon MSC and splenocyte co-culture, has been shown to upregulate SREBP-1c^51^, thereby increasing VLDL synthesis. On the other hand, IL-10 overexpression has been shown to result in reduced plasma cholesterol, mostly due to reduced VLDL, in LDLr KO mice^52^. Overall, our data indicates that MSCs reduce VLDL levels by decreasing inflammation in treated mice and thereby affecting hepatocytes and Kupffer cells.

Overall MSC therapy was highly effective in reducing both immune responses and improving dyslipidemia, the two driving forces behind atherosclerosis, and resulted in a significant 33% reduction in aortic root lesion sizes. Future studies will need to show the effect of MSCs on different stages of atherosclerosis, but our observations suggest that due to a combined effect on inflammation and plasma cholesterol levels a beneficial effect may be anticipated. For example, an increased stability of advanced lesions can be expected as IL-10 has been shown to promote lesion stability^53^,^54^. The fact that we do not observe an effect on lesion stability is directly associated with the early atherosclerotic lesions which contain a low amount of vascular smooth muscle cells. Recently, allogeneic MSCs were evaluated for their potential to repair ruptured lesions and were shown to increase regeneration of the inner endothelial lining and collagen fiber formation in the vessel wall^55^, implying their potential for the treatment of progressed lesions. Furthermore, a modulation of MSCs to enhance their anti-inflammatory capacities and/or their lifespan could prove interesting.

The drawback of some studies^10^,^11^ assessing the effect of MSCs on inflammation is that they employ human MSCs in SCID mice, which lack T and B cells. As these do not have a fully functional immune system it is difficult to examine the effect of MSCs on immune responses. Additionally, there have been reports that allogeneic MSCs can be rejected by recipient mice^10^, a caveat for a recent study by Lin *et al.*^56^. In this study human MSCs were transferred to ApoE KO mice, which increased eNOS in the aorta and thereby increased vasodilation in ApoE KO mice, but only resulted in a mild reduction of atherosclerosis. For these reasons, we performed an adoptive transfer of MHC-matched mouse MSCs into LDLr KO mice to avoid host immune responses to the grafted MSCs and to be able to do the experiment in mice with a functional immune system.

To translate our findings to clinical application, our studies could be in the future replicated using human MSCs in humanized mouse models for atherosclerosis, which can be humanized in their immune system, e.g. by transfer of human hematopoietic stem cells into SCID, NOG or NSG mice^57^,^58^. Also, a more humanized cholesterol metabolism, such as in the ApoE*3/CETP-Leiden mice^59^, would enable a confirmation of effects observed on VLDL metabolism. Taken together, our study provides evidence that a MSC-based treatment strategy has multifactorial therapeutic potential for inhibiting atherogenesis as it reduces inflammatory responses but also limits dyslipidaemia.

## Materials and Methods

### Animals

C57BL/6 and LDLr KO mice were originally obtained from the Jacksons Laboratory, kept under standard laboratory conditions, and administered food and water ad libitum. All animal work was approved by the Ethics Committee for Animal Experiments of Leiden University and conforms to Dutch government guidelines.

### DC culture

For DC cultures, bone marrow cells were isolated from the tibias and femurs of C57BL/6 mice. The cells were cultured for ten days at 37 °C and 5% CO_2_ in 10 cm^2^ petri dishes in IMDM supplemented with 10% FCS, 1% penicillin/streptomycin (all obtained from PAA), 1% glutamax (Thermo Fisher Scientific) and 20 μM β-mercaptoethanol (Sigma Aldrich) in the presence of 20 ng/mL granulocyte-macrophage colony-stimulating factor (Preprotech). DC purity was assessed by CD11c expression (flow cytometry) and routinely found to be above 95%.

### MSC isolation, phenotyping and labelling

Bone marrow cells were obtained by flushing femurs with washing medium (RPMI supplemented with 2% FCS, penicillin, streptomycin and L-glutamine). Next the bone marrow cells were cultured in a 75 cm^2^ flask containing αMEM (Life Technologies) supplemented with 10% FCS (Greiner Bio-One), 2% penicillin/streptomycin (Life Technologies) and 1% L-Glutamine (Life Technologies; MSC medium). Subsequently, plastic adherent MSCs were cultured to 95% confluency in a fully humidified atmosphere at 37 °C and 5% CO_2_, harvested using trypsin and further expanded until sufficient numbers were obtained.

The MSCs that were used throughout this study were of passage six to eight; for *in vivo* MSC treatment cells of passage eight were used. MSC were phenotyped using the following antibodies: TER119 (clone TER119), CD31 (clone MEC 13.3), CD45.2 (clone 104), CD90.2 (clone 53-2.1), CD29 (clone HMb1-1), Sca-1 (Clone D7), CD105 (clone MJ7/18), CD44 (clone IM7) and CD106 (clone 429). All antibodies were obtained from BD Biosciences.

For tracking experiments, MSCs were labelled with 10 μM Carboxyfluorescein succinimidyl ester (CFSE) according to manufacturer’s protocol (Thermo Fisher Scientific). Briefly, MSCs were resuspended in prewarmed PBS/0.1% BSA at a concentration of 1 × 10^6^ cells/mL and incubated with dye at 37 °C for 10 minutes in the dark. Afterwards, the staining was quenched by adding ice-cold media and incubating for another 5 minutes on ice, followed by three subsequent washing steps to remove excess CFSE.

### Co-cultures

For DC-MSC co-cultures, 1 million DCs were plated in 2 cm^2^ non-tissue culture treated petri dishes with indicated ratios of MSCs for 24 hours in MSC medium. DCs were stimulated with 100 ng/mL LPS to determine cytokine responses. Three independent experiments were done in triplicate.

For splenocytes-MSC co-cultures, single cell suspensions of spleens from LDLr KO mice were obtained by using a 70 μm cell strainer (VWR International). Red blood cells were lysed with erythrocyte lysis buffer (0.15 M NH_4_Cl, 10 mM NaHCO_3_, 0.1 mM EDTA, pH 7.2). 2 × 10^5^ splenocytes were added per well with indicated amounts of MSCs.

For T cell-MSC co-cultures, CD4^+^ T cells (>95% purity) were isolated from splenocytes by using the BD IMag^TM^ mouse CD4 T lymphocyte enrichment set according to manufacturer’s protocol (BD Biosciences). 1 × 10^5^ T cells were added per well with indicated amounts of MSCs.

Three independent experiments for both splenocytes and T cell-MSC co-cultures were cultured in quintuplicates in 96-well round-bottom plates in the presence or absence of αCD3/28 (2 μg/mL, eBioscience) for 72 hours in complete RPMI 1640, supplemented with 10% FCS, 100 U/ml penicillin/streptomycin, 2 mM L-glutamine (all obtained from PAA, Germany) and 20 μm β-mercaptoethanol (Sigma Aldrich). Proliferation was measured by Ki-67 expression by flow cytometry or addition of ^3^H-thymidine (0.5 μCi/well) for the last 16 hours of culture. The amount of ^3^H-thymidine incorporation was measured using a liquid scintillation analyzer (Tri-Carb 2900R) as the number of disintegrations per minute (dpm). T cell subsets were determined by flow cytometry.

We performed two independent experiments with splenocytes and T cell cultures in the presence of MSC culture supernatant in quintuplicates, MSC culture supernatant was added in different concentration of total medium added. MSC supernatant was filtered using a 0.2 μm filter to remove residual cells before use.

### Atherosclerosis

Atherosclerosis was induced in 16 weeks old male LDLr KO mice by feeding a Western-type diet (WTD) (0.25% cholesterol and 15% cocoa butter; Special Diet Services). 12 Mice per group received 3 *i.v.* injections of PBS or 0.5 million MSCs every other day prior to eight weeks WTD.

### Flow Cytometry

Mice were sacrificed and subsequently, blood and spleen were harvested of six mice per group. White blood cells were obtained as described above. 300,000 cells per sample were stained with the appropriate FACS antibodies. The following antibodies were used: CD11b-FITC (clone M1/70), CD11c-PE (clone N418), CD30L-PE (clone RM153), CD4-PerCP (clone RM4–5; BD Biosciences), CD40-FITC (clone HM40-3), CD8-PerCP (clone 53-6.7; BD Biosciences), CD80-PE (clone 16-10A1), CD86-APC (clone GL1), FoxP3-APC (clone FJK-16s), Gata-3-PE (clone TWAJ), Ki-67-FITC (clone SolA15), Ly-6G-APC (clone 1A8; BD Biosciences), MHCII-FITC (clone AF6-120.1), OX40L-PE (clone RM124L), PD-L2-APC (clone MIH18), RORγt-PE (clone AFKJS-9), and T-bet-Alexa Fluor 647 (clone eBio4B10). All antibodies were purchased from eBioscience, unless stated otherwise. For intracellular staining, cells were fixed and permeabilized according to the manufacturer’s protocol (eBioscience). FACS analysis was performed on the FACS Canto II and data were analyzed using FACS Diva software (BD Biosciences).

### *In Vivo* Imaging Systems (IVIS)

For MSC tracking experiments, 1 × 10^6^ MSCs were stained with CFSE and injected *intravenously. Ex vivo* imaging was performed by placing either whole animals or organs in the IVIS Lumina Imaging System (Xenogen) at indicated time points and analyzing fluorescence based on the manufacturer’s recommendations. Fluorescence intensity was quantified as photons/sec/cm^2^ by Living Image software (Xenogen).

### Histological analysis

To determine plaque size, 10 μm cryosections of the aortic root of all mice were stained with Oil-Red-O and haematoxylin (Sigma Aldrich). Corresponding sections were stained for collagen fibers using the Masson’s Trichrome staining (Sigma Aldrich) or immunohistochemically with antibodies against a macrophage specific antigen (MOMA-2, polyclonal rat IgG2b, 1:1000, Serotec Ltd.). Goat anti-rat IgG alkaline phosphatase conjugate (dilution 1:100; Sigma Aldrich) was used as a secondary antibody and nitro blue tetrazolium and 5-bromo-4-chloro-3-indolyl phosphate as enzyme substrates. To determine the number of adventitial T cells, CD3 staining was performed using anti-mouse CD3 (clone SP7, 1:150, ThermoScientific). BrightVision anti-rabbit-HRP was used as secondary antibody (Immunologic). The section with the largest lesion and four flanking sections were analyzed for lesion size and collagen content, two flanking sections were analyzed for macrophage and T cell content. All images were analyzed using the Leica DM-RE microscope and LeicaQwin software (Leica Imaging Systems, UK). The percentage of collagen, macrophages and Oil-Red-O in the lesions was determined by dividing the collagen, MOMA-2- or Oil-Red-O positive area by the total lesion surface area. The plaque percentage was calculated by dividing plaque size by the total root area (i.e. plaque + lumen size). Relative cap area (in %) was calculated by measuring cap size as percentage of total plaque size.

### Real-time PCR

mRNA was isolated from the liver, draining lymph nodes of the heart and white adipose tissue of all mice using the guanidium isothiocyanate method and reverse transcribed (RevertAid Moloney murine leukemia virus reverse transcriptase). Quantitative gene expression analysis was performed on a 7500 Fast real-time PCR system (Applied Biosystems) using SYBR Green technology. The expression was determined relative to the average expression of three housekeeping genes: succinate dehydrogenase complex, Subunit A (SDHA), hypoxanthine phosphoribosyltransferase (HPRT), and 60S ribosomal protein L27 (Rpl27). For used primer pairs please refer to Supplementary Table 1.

### Cytokines

IL-10, TNF-α, IFN-γ in cell culture supernatant were determined by ELISA (BD Biosciences), while IL-10, TNF-α, IFN-γ serum levels were determined in 3–6 animals per group by luminex immunoassay (eBioscience, procartaplex) according to manufacturer’s protocol. CCL2 and IL-6 levels in serum were determined in all mice by ELISA (BD Biosciences) according to manufacturer’s protocol.

### Serum cholesterol levels

During the experiment, mice were weighed and blood samples were obtained by tail vein bleeding. Serum concentrations of total cholesterol were determined by enzymatic colorimetric assays (Roche Diagnostics). Precipath (standardized serum; Roche Diagnostics) was used as internal standard. The distribution of cholesterol over the different lipoproteins in serum was determined by fractionation of 30 μl of serum using a Superose 6 column (3.2 × 300 mm, Smart-System; Pharmacia). Total cholesterol content of the effluent was determined as described above.

### Statistical analysis

Values are expressed as mean ± SEM and were tested for normal distribution. Data of two groups were analyzed with a two-tailed Student’s T-test. Data of three or more samples were compared by one-way ANOVA and data of two groups with more than one variable were analyzed by two-way ANOVA, both followed by Bonferroni post-testing. Statistical analysis was performed using Prism (GraphPad). Probability values of P < 0.05 were considered significant.

## Additional Information

**How to cite this article**: Frodermann, V. *et al.* Mesenchymal Stem Cells Reduce Murine Atherosclerosis Development. *Sci. Rep.* **5**, 15559; doi: 10.1038/srep15559 (2015).

## Supplementary Material

Supplementary Information

## Figures and Tables

**Table 1:** MSCs only mildly affect co-stimulatory molecule expression of LPS-stimulated DCs.

	**DCs only**	**½ MSC per DC**	**1 MSC per DC**	**2 MSCs per DC**
Percentages				
CD80	95.5 ± 0.3	97.5 ± 0.4	98.2 ± 0.1	98.2 ± 0.1
CD86	68.1 ± 0.7	73.8 ± 0.3 (***)	76.0 ± 0.1 (***)	76.5 ± 0.1 (***)
CD40	88.9 ± 0.3	92.1 ± 2.0	90.1 ± 0.8	93.43 ± 0.2
OX40L	35.5 ± 0.8	47.6 ± 3.7 (*)	43.2 ± 1.1	44.1 ± 0.4
CD30L	18.2 ± 1.9	17.6 ± 12	19.5 ± 1.2	21.1 ± 1.5
PD-L2	50.9 ± 0.6	56.5 ± 2.3	61.8 ± 0.9 (**)	62.5 ± 0.15 (**)
MFI				
CD80	12666 ± 118	15568 ± 407 (***)	16351 ± 321 (***)	16961 ± 272 (***)
CD86	49906 ± 446	55290 ± 957 (***)	52075 ± 55	49102 ± 75
CD40	12447 ± 177	12065 ± 1148	11825 ± 287	12622 ± 12
OX40L	7439 ± 78	7655 ± 221	8196 ± 139	8135 ± 91
CD30L	2943 ± 41	2953 ± 53	2924 ± 32	3018 ± 59
PD-L2	5469 ± 105	5012 ± 100	4695 ± 211	4534 ± 76 (*)

DCs were co-cultured with indicated ratios of MSCs for 3 hours prior to addition of 100 ng/mL LPS for 24 hours. DC numbers remained constant. Co-stimulatory molecules on DCs, determined as CD11c^+^MHCII^+^, were determined by flow cytometry. The percentage and mean fluorescence intensity (MFI) is shown. All values are expressed as mean ± SEM and representative of two independent experiments done in triplicate. *P < 0.05, **P < 0.01, ***P < 0.001.
