# Supplementary material for: Mesenchymal Stem Cells Reduce Murine Atherosclerosis Development
Source: Sci Rep. 2015 Oct 22;5:15559. doi: 10.1038/srep15559 (PMC4614841; doi:10.1038/srep15559)
Supplement: Supplementary Information [file srep15559-s1.pdf]

Supplementary Information

Mesenchymal Stem Cells Reduce Murine Atherosclerosis Development

Frodermann V, van Duijn J, van Pel M, van Santbrink PJ, Bot I, Kuiper J, de Jager SCA

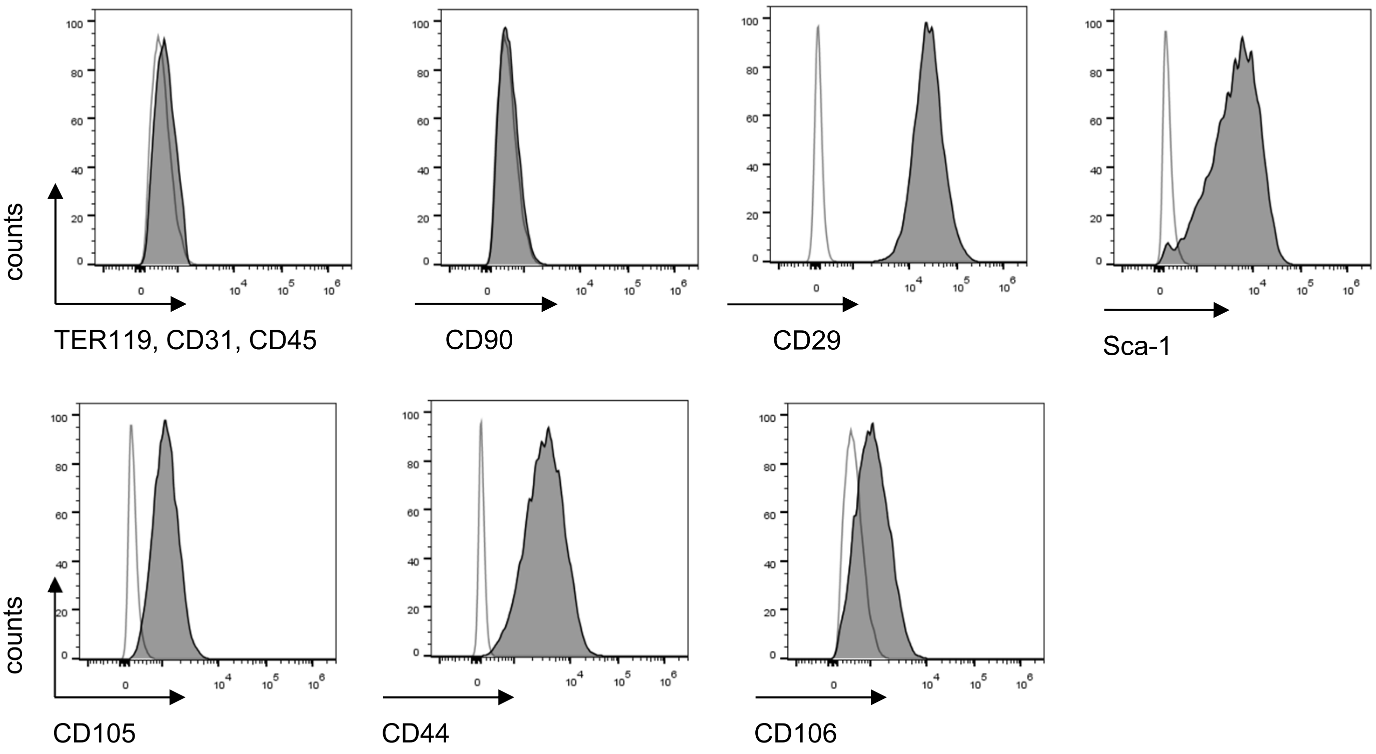

Supplementary Figure 1. Phenotype of MSCs. MSCs were generated from the bone marrow of male C57BL/6 mice and the expression of surface markers was analyzed by flow cytometry. Representative histograms are shown.

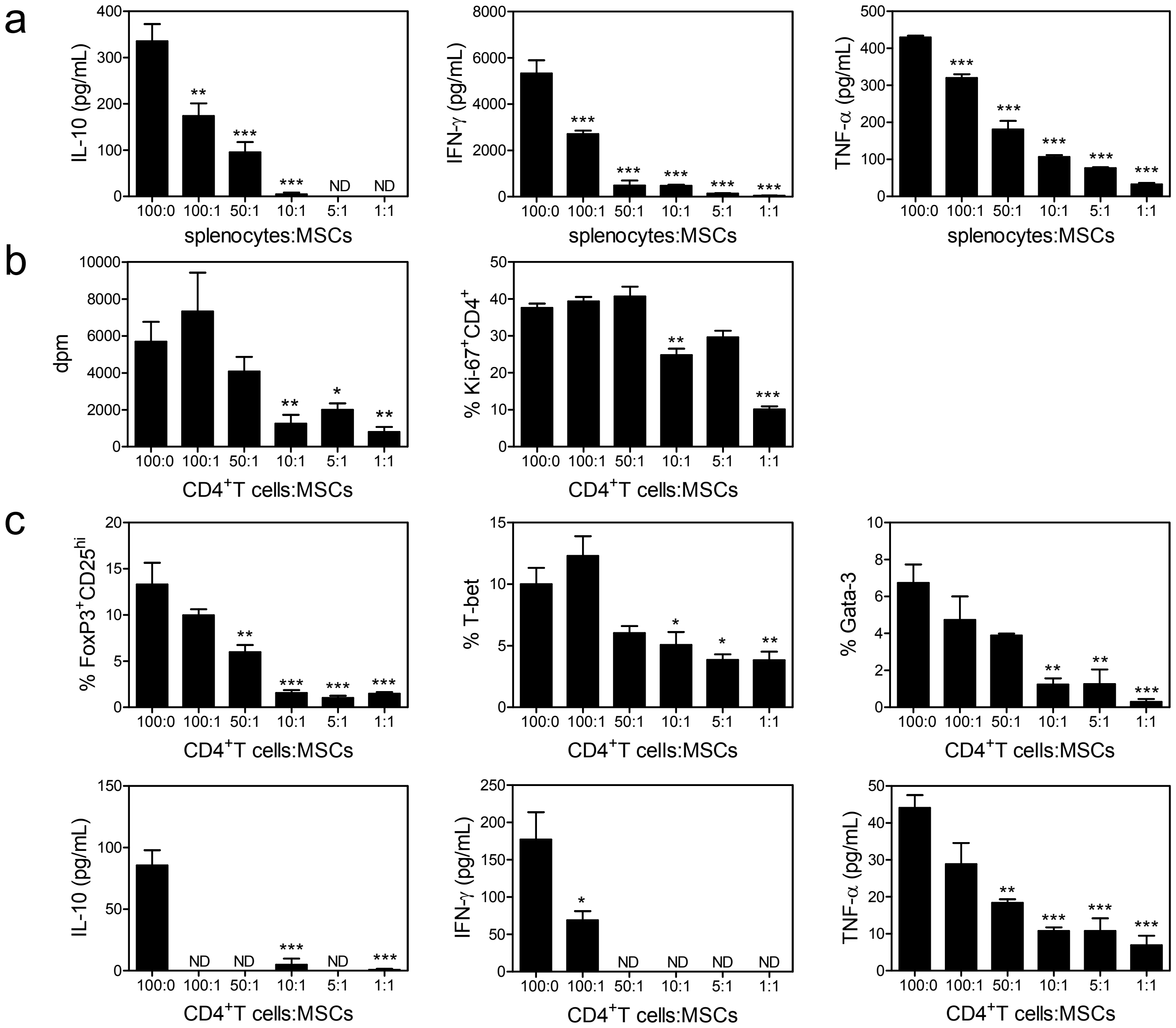

Supplementary Figure 2. MSCs reduce T cell proliferation *in vitro.* Total splenocytes and splenic CD4⁺ T cells, both obtained from LDLr KO mice, were co-cultured with indicated ratios of MSCs in the presence of αCD3/CD28 for 72 hours. Splenocyte and T cell numbers remained constant. a, Splenocytes showed reduced cytokine production in the presence of MSCs. b, Proliferation of $CD4^+$ T cells was assessed by ^{3}H-thymidine and by Ki-67 expression by flow cytometry. c, T cell subsets within $CD4^+$ T cells were determined by flow cytometry. Cytokine production was determined by ELISA. All values are expressed as mean±SEM and representative of at least two independent experiments done in triplicate. * P<0.05, ** P<0.01, *** P<0.001. ND defines not determined.

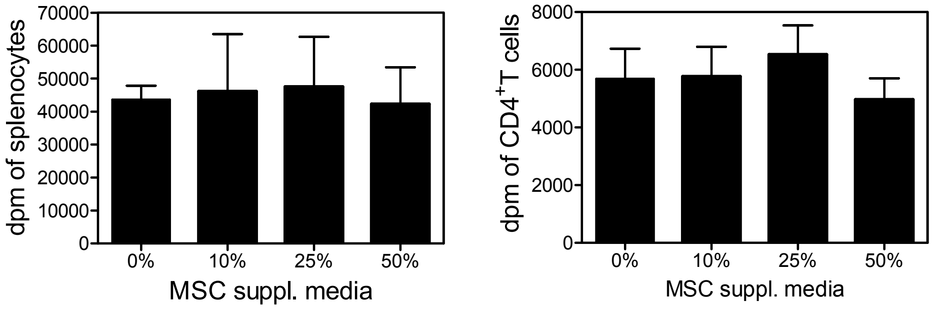

Supplementary Figure 3. MSC supernatant does not affect T cell proliferation. Splenocytes and $CD4^+$ T cells obtained from LDLr KO mice were cultured in the presence of MSC culture supernatant at the indicated amount in the presence of αCD3/28 for 72 hours. Proliferation was assessed by ^{3}H-thymidine incorporation. Splenocyte and T cell numbers remained constant. All values are expressed as mean±SEM and representative of two independent experiments done in triplicate.

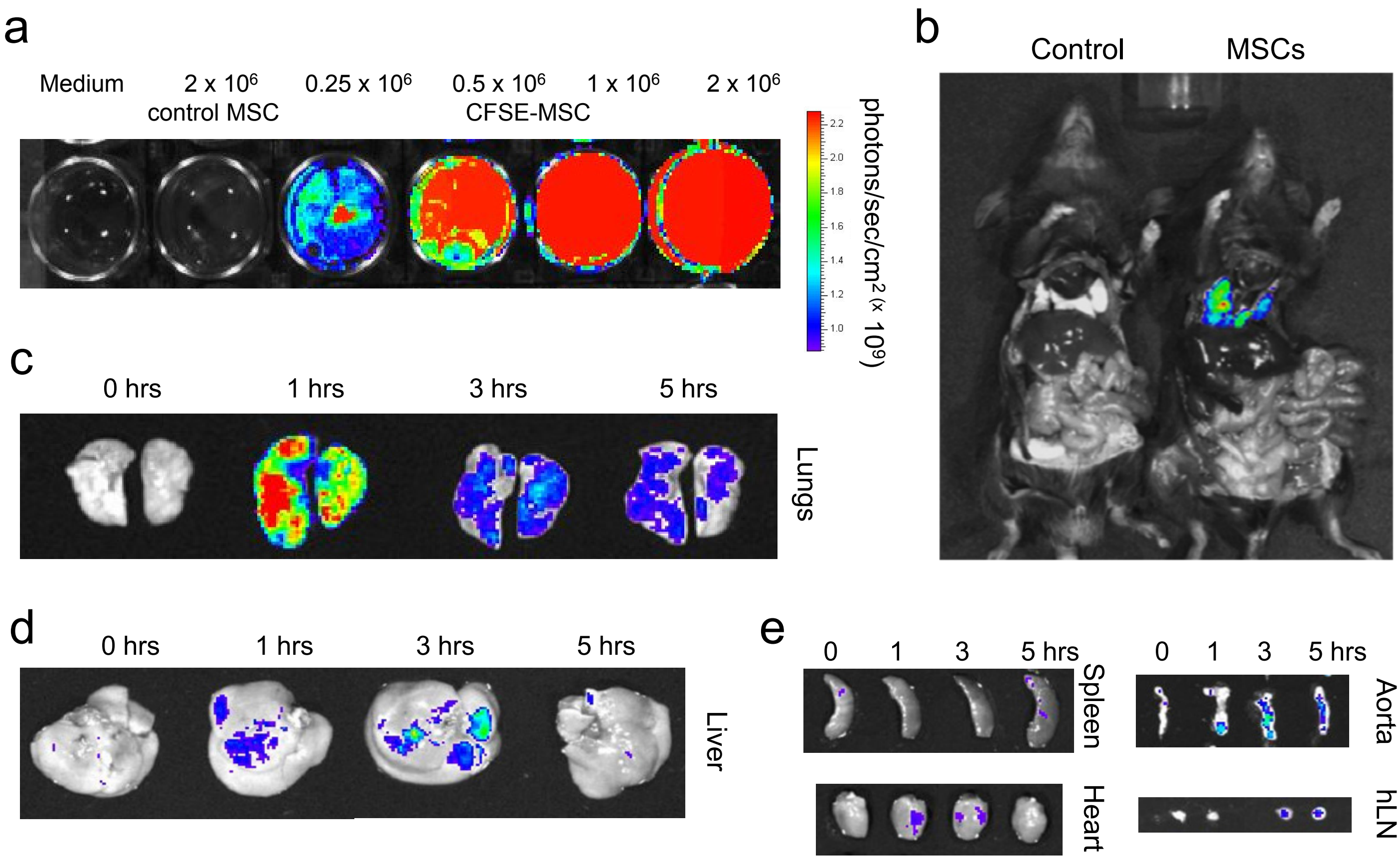

Supplementary Figure 4. CFSE-labelled MSCs migrate preferentially to lung, liver, and the vasculature. a, MSCs were labelled with 10μM CFSE. Signal intensity on IVIS correlates with amount of cultured $CFSE^+$ MSCs. Control MSCs indicates non-labelled MSCs. b, 15 min after *i.v.* injection of 1 x 10^6 MSCs into LDLr KO, MSCs accumulate in the lung as determined by IVIS. c, MSC presence in the lung 1-5 hrs after injections into LDLr KO mice one week on WTD. d, liver and e, organ distribution of MSCs as determined by IVIS 1-5 hrs after injection of MSCs into LDLr KO mice one week on WTD.

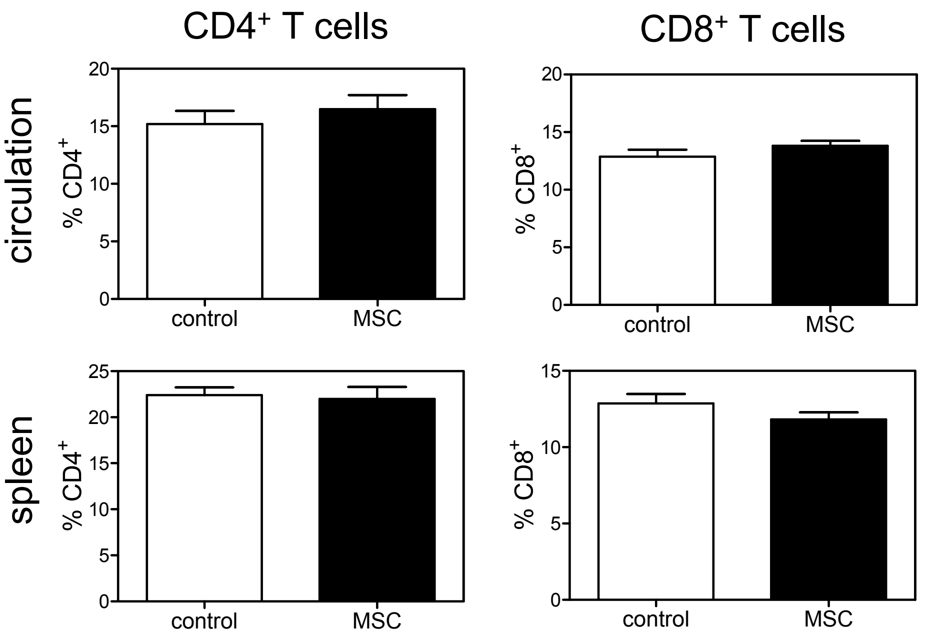

Supplementary Figure 5. MSC-treatment does not affect absolute CD4$^+$ and CD8$^+$ T cell numbers *in vivo*. Male LDLr KO mice received three *i.v.* injections of either PBS (control) or 0.5 x 10^6 MSCs (MSC) and were then fed a Western-type diet (WTD) for eight weeks. CD4$^+$ and CD8$^+$ T cells percentages were determined in the circulation and the spleen by flow cytometry. All values are expressed as mean±SEM and representative of six mice.

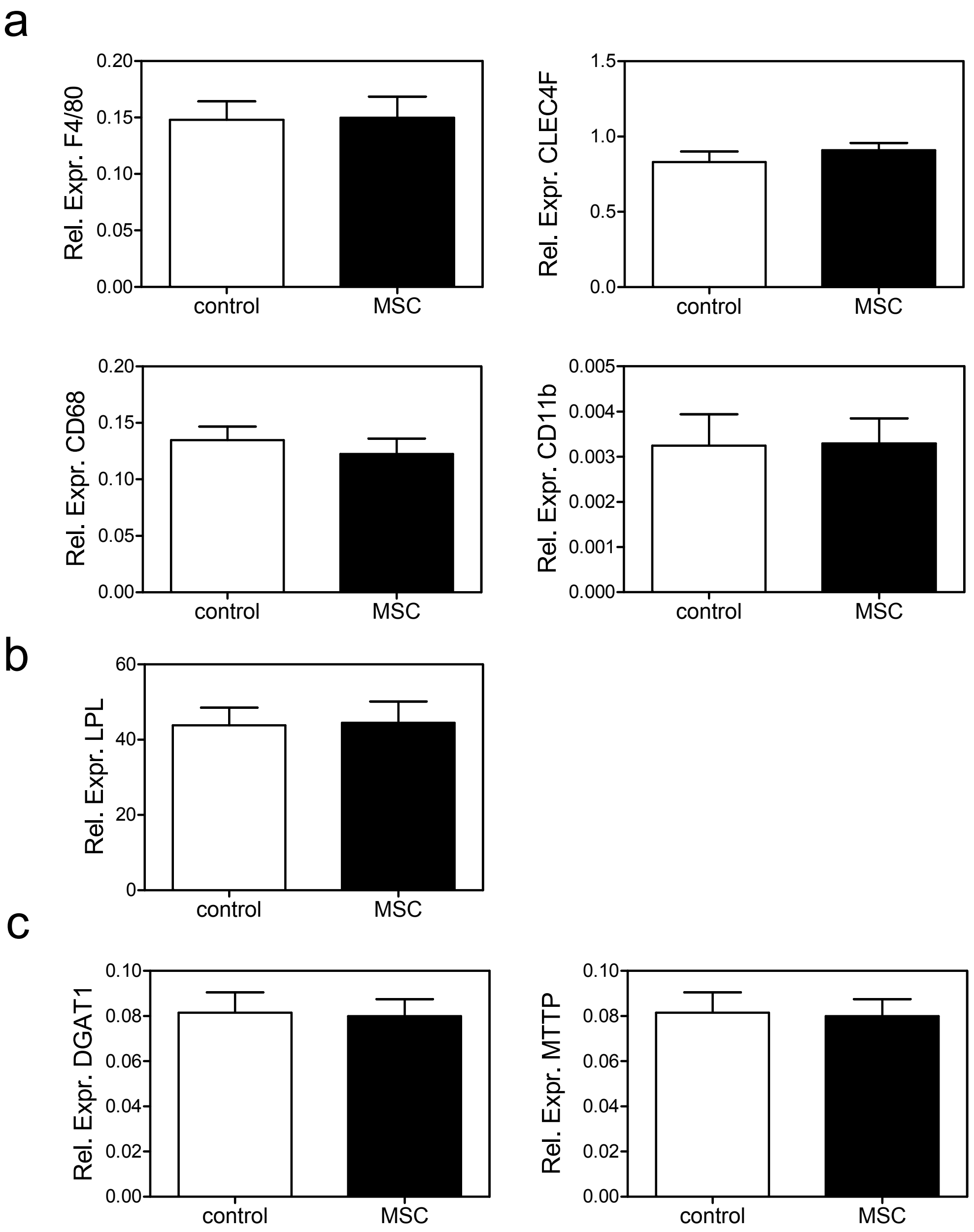

Supplementary Figure 6. MSC treatment effect on mRNA expression in the liver. a, Liver mRNA expression of Clec4f, F4/80, CD11b, and CD68 is shown. b, White adipose tissue mRNA expression of lipoprotein lipase (LPL) is shown. c, Liver mRNA expression of diglyceride acyltransferase 1 (DGAT1) and microsomal triglyceride transfer protein (MTTP), relative to the expression of three housekeeping genes (SDHA, HPRT and Rpl27). All values are expressed as mean±SEM and representative of all mice. *P<0.05, **P<0.01, ***P<0.001.

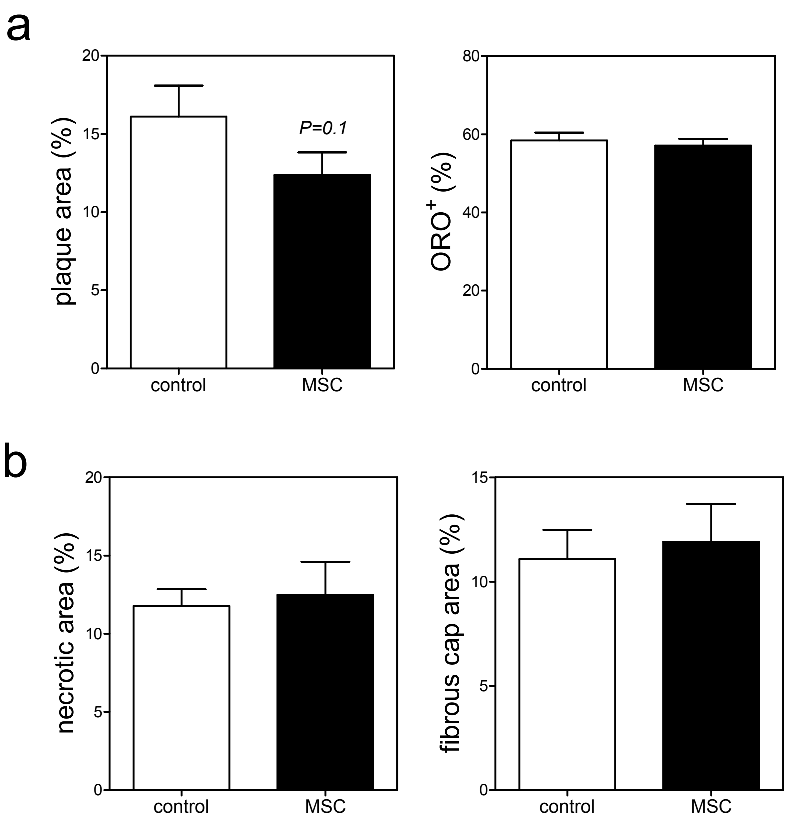

Supplementary Figure 7. MSC treatment reduces lesion development but does not affect lesion composition. a, The % plaque area was determined as total lesion area divided by total area of lumen and lesion. Lipid deposition was assessed by measuring Oil-red O positive area of lesions. b, necrotic area was determined as percentage acellular space of lesions and fibrous cap size as cap area divided by total lesion area.

Gene	*Forward*	*Reverse*
**ACC1**	AGAATCTCCTGGTGACAATGCTTATT	GCTCTGTGAGGATATTTAGCAGCTC
**CD68**	TGCCTGACAAGGGACACTTCGGG	GCGGGTGATGCAGAAGGCGATG
**CLEC4F**	AGATCTGCAGGCGACCAAAGCC	TCTGCGCGATCAGCTGGAGAAC
**DGAT1**	GGTGCCCTGACAGAGCAGAT	CAGTAAGGCCACAGCTGCTG
**F4/80**	TGCAGGGGGCTTTCATCTTCCTCA	GGGAGCTAAGGTCAGTCTTCCTGGTG
**FASN**	GGCGGCACCTATGGCGAGG	CTCCAGCAGTGTGCGGTGGTC
**HPRT**	TACAGCCCCAAAATGGTTAAGG	AGTCAAGGGCATATCCAACAAC
**IL-6**	AGACAAAGCCAGAGTCCTTCAGAGA	GGAGAGCATTGGAAATTGGGGTAGG
**IL-10**	GGGTGAGAAGCTGAAGACCCTC	TGGCCTTGTAGACACCTTGGTC
**LPL**	CCCCTAGACAACGTCCACCTC	TGGGGGCTTCTGCATACTCAAA
**MTTP**	TCTCACAGTACCCGTTCTTGGT	GAGAGACATATCCCCTGCCTGT
**RPL27**	CGCCAAGCGATCCAAGATCAAGTCC	AGCTGGGTCCCTGAACACATCCTTG
**Scd1**	TACTACAAGCCCGGCCTCC	CAGCAGTACCAGGGCACCA
**SDHA**	TATATGGTGCAGAAGCTCGGAAGG	CCTGGATGGGCTTGGAGTAATCA
**SREBP-1C**	TCTGAGGAGGAGGGCAGGTTCCA	GGAAGGCAGGGGGCAGATAGCA
**SREBP-2**	TGAAGCTGGCCAATCAGAAAA	ACATCACTGTCCACCAGACTGC
**TNF-α**	GCCTCTTCTCATTCCTGCTTGTG	ATGATCTGAGTGTGAGGGTCTGG

Supplementary Table 1. Primer Pairs used for qPCR analysis. The relative expression of genes was determined relative to the average expression of three housekeeping genes: hypoxanthine-guanine phosphoribosyltransferase (HPRT), succinate dehydrogenase complex, Subunit A (SDHA) and ribosomal protein L27 (Rpl27). Abbreviations: ACC1, Acetyl-CoA carboxylase 1; DGAT1, diglyceride acyltransferase 1; FASN, fatty acid synthase; LPL, lipoprotein lipase; MTTP, microsomal triglyceride transfer protein; Scd1; stearoyl-CoA desaturase-1; SREBP-1c, sterol regulatory element-binding protein 1c; SREBP-2, sterol regulatory element-binding protein 2.
